# Production system influences color stability and lipid oxidation in *gluteus medius* muscle

**DOI:** 10.5713/ab.22.0271

**Published:** 2022-11-14

**Authors:** Ana Paula Amaral de Alcântara Salim, Micheli da Silva Ferreira, Maria Lucia Guerra Monteiro, Loíse Caroline Santos de Lima, Isabelle Trezze Marins Magalhães, Carlos Adam Conte-Júnior, Sérgio Borges Mano

**Affiliations:** 1 Department of Food Tecnology, Faculty of Veterinary Medicine, Fluminense Federal University (UFF), Niterói, RJ 24230-340, Brazil; 2 Center for Food Analysis (NAL), Technological Development Support Laboratory (LADETEC), Federal University of Rio de Janeiro, Rio de Janeiro, RJ 21941598, Brazil; 3 Chemistry Institute, Federal University of Rio de Janeiro, Rio de Janeiro, RJ 21941909, Brazil; 4 Graduate Program in Chemistry (PGQu), Chemistry Institute, Federal University of Rio de Janeiro, Rio de Janeiro, RJ 21941909, Brazil; 5 Analytical and Molecular Laboratorial Center (CLAn), Institute of Chemistry (IQ), Federal University of Rio de Janeiro (UFRJ), Cidade Universitária, Rio de Janeiro, RJ, 21941-909, Brazil; 6 Department of Biochemistry, Chemistry Institute, Federal University of Rio de Janeiro, Rio de Janeiro, RJ 21941598, Brazil; 7 National Institute of Health Quality Control, Oswaldo Cruz Foundation (FIOCRUZ), Rio de Janeiro, RJ 21040900, Brazil

**Keywords:** Beef Discoloration, Feeding Regime, Instrumental Color, Metmyoglobin Reducing Activity (MRA), Oxidative Stability, Top Sirloin

## Abstract

**Objective:**

We aimed to evaluate the color and oxidative stability of beef *gluteus medius* (GM) from cattle raised in organic and non-organic production systems.

**Methods:**

The GM samples (n = 10) were obtained from organic (ORG; n = 5) or non-organic (NORG; n = 5) beef samples, sliced into 2.54-cm steaks, packaged in aerobic conditions, and stored for nine days at 4°C. ORG and NORG steaks were compared regarding myoglobin concentration, pH, instrumental color, delta E (ΔE), metmyoglobin reducing activity (MRA), and lipid oxidation on days 0, 5, and 9.

**Results:**

Feeding system did not influence (p>0.05) the myoglobin concentration. ORG steaks exhibited greater (p<0.05) meat pH, yellowness, and MRA, whereas NORG steaks exhibited greater (p<0.05) redness, chroma, R630/580, delta E, and lipid oxidation. ORG and NORG steaks exhibited similar (p>0.05) lightness and hue angle. During storage, ORG and NORG exhibited an increase in muscle pH, hue angle, and lipid oxidation; and a decrease (p<0.05) in redness, yellowness, chroma, and color stability (R630/580). Both samples exhibited a stable (p>0.05) pattern for lightness and MRA.

**Conclusion:**

Therefore, the production system can affect beef color and lipid stability during storage.

## INTRODUCTION

Brazil is the major producer and exporter of beef, with most livestock production based on pasture feeding (constituted of pasture; pasture plus concentrate; grass; forage and silage-based). In addition to pasture, the feedlot grain-based system has been widely used to limit cattle weight loss, especially during the dry season [1]. In this context, the beef from alternative raising systems, such as organic pasture feeding, has been standing out due to the consumer demand for products with production and processing quality attributes, and free of chemical residues and contaminants [2]. Beef consumption represents 40% of total animal protein consumed in Brazil, supporting the importance of beef to the economy of the whole country [1].

Meat color is the main attribute influencing purchase decisions and is governed by a multitude of intrinsic and extrinsic factors. The desired color of fresh beef is bright cherry red, and any deviation in its appearance leads to consumer rejection and economic losses [3]. The rearing system has been associated with development of meat quality characteristics [4,5]. Nonetheless, investigations in this aspect have demonstrated conflicting results.

Cozzi et al [4] documented a lower lightness and greater redness and yellowness in organic beef from *Bos taurus* cattle than those from non-organic counterparts. Ferraz et al [6] reported greater lightness, redness, and yellowness in meat from organic Nellore cattle than their feedlot-raised counterparts. On the other hand, Bjorklund et al [5] investigated the influence of the feeding systems organic vs. conventional (80% concentrate plus 20% forage) and observed lower redness in organic beef from Holstein and crossbred steers when compared to their non-organic counterparts.

Although previous investigations have demonstrated the influence of feeding systems on the color of *Bos taurus* [4,5] and *Bos indicus* [6] cattle, the influence of the production system on color stability and lipid oxidation of beef from Nellore cattle is yet to be investigated. Therefore, the aim of the present study was to evaluate the influence of the production system (organic vs non-organic) on beef color stability, and lipid oxidation during 9 days of refrigerated storage.

## MATERIALS AND METHODS

### Experimental design and beef fabrication

The *gluteus medius* (GM) samples were purchased from a commercial facility and transported to Universidade Federal Fluminense (Niteroi, Rio de Janeiro, Brazil). Therefore, institutional animal care and use committee approval was not obtained. Ten (n = 10) vacuum packaged GM muscles (NAMP #184) from pasture-fed organic (ORG; n = 5) and grain-finished non-organic (NORG; n = 5) production systems were used in this experiment. The GM were purchased from the same producer, and information regarding the production systems was obtained from the product label and manufacturer websites. ORG samples were obtained from organic pasture-fed cattle, which production was certified by the Instituto Biodinâmico de Desenvolvimento Rural (IBD) Certification Agency, affiliated to the International Federation of Organic Agriculture Movements. All the ingredients offered to animals were organic and free of urea for 30 to 36 months until the harvest. NORG samples were obtained from non-organic grain-finished cattle with diet composed of 80% corn, during 90 days before harvest.

After purchase, the samples were transported under re frigeration (4°C) to the Universidade Federal Fluminense. All external fat was removed, and the GM muscles were fabricated into six 2.5-cm thick steaks. The steaks were individually packaged on polystyrene trays with soaker pads, over-wrapped with oxygen-permeable polyvinyl chloride film (0.014 mm thickness; 15,500 to 16,275 cm^3^/m^2^/24 h oxygen transmission rate at 23°C), and assigned randomly for 0, 5, and 9 days at 4°C. On day 0, two steaks were assigned for analyses of myoglobin concentration, pH, instrumental color, metmyoglobin reducing activity (MRA), and lipid oxidation. The remained four steaks were utilized to evaluate instrumental color and biochemical attributes on days 5 and 9 (two steaks/d; 1 for color and 1 for biochemical analyses). All the analyses were performed in duplicate.

### Myoglobin concentration

The myoglobin (Mb) concentration was determined according to Faustman and Phillips [7]. Samples of 5 g were homogenized with 45 mL ice-cold sodium phosphate buffer (40 mM, pH 6.8) and centrifuged (5,000×g at 4°C for 90 min). Additionally, the supernatant was filtered using Whatman no. 1 paper, and the absorbance of the filtrate at 525 nm (A525) was recorded using a UV-1800 spectrophotometer (Shimadzu Corporation, Kyoto, Japan). The Mb concentration was calculated using the following equation:


$$\text{Myoglobin }(\text{mg}/\text{g muscle tissue})=[\text{A}525/(7.6\;{\text{mM}}^{-1}{\text{cm}}^{-1}\times 1\;\text{cm})]\times (17,000/1,000)\times 10$$

Where: 7.6 mM^−1^cm^−1^ = mM absorptivity coefficient of Mb at 525 nm; 1 cm = light path length of cuvette; 17,000 Da = average molecular weight of Mb; 10 = dilution factor.

### Meat pH

The pH was measured utilizing a portable pH meter (ISTM Instruments, Rio de Janeiro, Brazil) equipped with an insertion-type probe [8].

### Instrumental color evaluation

The surface lightness (L*), redness (a*), and yellowness (b*) values were measured using a portable spectrophotometer CM-600D (Konica Minolta Sensing Inc., Osaka, Japan) equipped with illuminant A, 8 mm aperture, and 10° standard observer (AMSA [9]). The color was measured at three random locations on the steak surfaces. Color stability was indirectly estimated through the ratio of reflectance at 630 nm and 580 nm (R630/580) according to AMSA [9]. Additionally, were determined the absolute differences in color coordinates (Deltas; Δ) between the days 0 and 9. Deltas for L* (ΔL*), a* (Δa*), and b* (Δb*) were expressed as negative (−) or positive (+) results, whereas the total color difference or Delta E (ΔE) was only positive. The Deltas were expressed as ΔL* = difference between lighter and darker (+ = lighter; − = darker); Δa* = differences between red and green (+ = redder; − = greener); Δb* = differences between yellow and blue (+ = yellower, − = bluer). The total color change or Delta E (ΔE), was calculated using the average of initial color readings (day 0) and the final readings (day 9), according to AMSA [9]:


$$\Delta \text{E}=[{\left(\Delta L^{★}\right)}^{2}+{\left(\Delta a^{★}\right)}^{2}+{\left(\Delta b^{★}\right)}^{2}]1/2.$$

### Metmyoglobin reducing activity

Metmyoglobin reducing activity was evaluated according to the method of Sammel et al [10]. Two cubes (2.0 cm×2.0 cm ×2.0 cm) were sliced from each steak and individually submerged in sodium nitrite solution (0.3%) for 20 minutes to induce metmyoglobin formation. Then, the cubes were blotted dry, vacuum-packed, and the reflectance values (from 400 to 700 nm) were recorded using a portable spectrophotometer CM-600D (Konica Minolta Sensing Inc., Japan). The samples were incubated at 30°C for 2 hours to allow the metmyoglobin reduction and the previously evaluated surfaces were rescanned. Metmyoglobin formation on the surface was calculated utilizing the absorption coefficient/scattering coefficient (K/S) ratios and formulas according to AMSA [9], and the MRA was estimated using the following equation:


$$\begin{array}{l} \text{MRA}=100\times [(\text{pre-incubation percentage of surface metmyoglobin})-(\text{post-incubation percentage of}\\ \text{surface metmyoglobin)}]/[(\text{pre-incubation percentage of surface metmyoglobin})] \end{array}$$

### Lipid oxidation

Lipid oxidation was evaluated according to Sinnhuber et al [11] and Buege and Aust [12]. Samples (5 g) were homogenized with 22.5 mL trichloroacetic acid solution (TCA; 11%) and centrifuged (11,000×g at 4°C for 15 min). One milliliter of the supernatant was mixed with 1 mL of an aqueous solution of thiobarbituric acid (20 mM) and incubated at 25°C for 20 h. The absorbance values at 532 nm were measured using a UV-1800 spectrophotometer (Shimadzu Corporation, Japan) and presented as thiobarbituric acid reactive substances (TBARS).

### Statistical analysis

This study utilized ten (n = 10) beef carcasses, and the experimental design was completely randomized. Data were analyzed using XLSTAT software (Version 2014.5.03; Addinsoft, Inc., Brooklyn, NY, USA). One-way analysis of variance (ANOVA) was used to analyze Mb concentration. A two-way ANOVA was utilized to analyze pH, instrumental color, MRA, and lipid oxidation to assess the effect of the feeding system (organic vs non-organic) and days of storage (0, 5, and 9). Tukey’s test was used to compare treatment means at a 5% significance level (p<0.05). The results of the physicochemical analyses were also evaluated by principal components analysis (PCA) and Pearson’s correlation.

## RESULTS AND DISCUSSION

### Myoglobin concentration

The feeding system did not influence (p>0.05) the Mb concentration of steaks (5.33±0.34 mg/g in ORG; 5.91±0.54 mg/g in NORG), which could be attributed to muscle fiber type [13]. The muscle fibers can be divided into three major categories: type I, slow-twitch oxidative; type IIA, fast-twitch oxidative glycolytic; and type IIB, fast-twitch glycolytic. For instance, color-stable muscle (i.e., *longissimus lumborum*; LL) is composed of fiber IIB, whereas color-labile muscles (i.e., *psoas major*; PM) are composed mainly of type I fiber, influencing myoglobin concentration [13]. *Gluteus medius* is classified as an intermediate color stability muscle [14] composed of fiber type I, IIA, and IIB and exhibited intermediate values of myoglobin concentration when compared to LL and PM muscles. This in turn, may have contributed to the similarity in Mb concentration observed in ORG and NORG samples.

In contrast with our results, Apaoblaza et al [15] reported greater myoglobin content in meat from pasture-fed steers than those from feedlot-fed. Torrecilhas et al [16] documented higher myoglobin content in *longissimus thoracis* muscle from pasture-fed cattle than their counterparts obtained from feedlot (25:75% corn silage: concentrate).

### Meat pH

There was a production system×storage interaction (p = 0.002; Table 1) for meat pH. The ORG steaks exhibited greater meat pH (p<0.05) than NORG on days 0 and 5 (Table 1), whereas ORG and NORG steaks exhibited similar (p>0.05) pH values on day 9 of storage. The observed differences in pH between NORG and ORG steaks could be possibly attributed to differences in muscle energy and metabolism occasioned by feeding strategy [15]. Apaoblaza et al [15] investigated the influence of the feeding system on changes in muscle characteristics and postmortem metabolism and documented that muscle from grass and grain-fed cattle differs energetically. Grain-fed cattle exhibited higher content of enzymes involved in muscle glycolytic metabolism (as lactate dehydrogenase and glyceraldehyde phosphate dehydrogenase) than grass-fed cattle [15], which in turn contributes to the increase of lactate content and decrease of pH values in muscle as observed in NORG samples.

Our results agree with Revilla et al [17], which evaluated the influence of the production system (conventional vs organic) on beef quality and documented a lower pH value in beef from pasture plus grain-fed from Limousin×Avileña calves when compared to their pasture-fed counterparts. Apaoblaza et al [15] documented lower pH values in *longissimus dorsi* muscle from grain-fed cattle than those from pasture-fed counterparts. Contrasting our results, Cozzi et al [4] evaluated the meat quality parameters of organic beef from *Bos taurus* cattle and observed a lower pH in organic beef from pasture-finished cattle than in their grain-finished counterparts.

Both ORG and NORG samples exhibited stable (p >0.05) pH values during storage. In contrast with our results, Revilla et al [17] documented a decrease in pH values in beef from conventional and organic raising systems during 14 days of storage. Fruet et al [18] reported an increase in pH values in beef from concentrate and legume-grass steers from day 0 to 13 of storage.

### Lightness (L* value)

There was no production system×storage interaction (p = 0.466; Table 1) for L* value. Also, there was no effect of production system (p = 0.082) and storage (p = 0.455) on lightness. The ORG and NORG steaks exhibited similar (p>0.05) lightness (L* values) on days 0, 5, and 9 of storage (Table 1). The observed similarity in L* values between ORG and NORG steaks could be attributed to the muscle fiber composition of GM [14]. *Gluteus medius* is an intermediate muscle composed of muscle fiber types I, IIA, and IIB, demonstrating greater lightness than muscles composed of fiber type I [14]. The glycolytic potential of fiber IIB favors the use of glycogen as an energy source contributing to a rapid postmortem pH decrease, which influences the light reflectance, affecting L* values [19]. In this sense, using GM samples for both ORG and NORG samples may have contributed to the similar lightness.

In agreement, Bjorklund et al [5] evaluated the sensory attributes of beef from organic and conventional (80% concentrate plus 20% forage) raised Holstein and crossbred steers and observed similar lightness in beef from organic and non-organic cattle. In addition, Fruet et al [18] reported similar L* values in beef from steers finished with concentrate and legume-grass pasture on days 0 and 13 of storage. In partial agreement, Kim et al [20] evaluated the influence of feeding regimes (pasture grazing and barn feeding) on meat quality of elk deer loin and documented similar lightness on days 0 and 14 of refrigerated storage. In contrast, Torrecilhas et al [16] documented a lower lightness in beef from pasture-fed cattle than in their feedlot counterparts. Apaoblaza et al [15] reported higher lightness (L* values) in *longissimus dorsi* from grain-fed than those values of beef from grass-fed cattle. Cozzi et al [4] evaluated meat quality parameters of beef from *Bos taurus* cattle finished with pasture grazing or grains and observed a lower lightness in beef from pasture-finished cattle. Also, a darker meat color that results from a lower lightness has been reported in beef from grass-fed cattle [21].

Regarding storage, both ORG and NORG exhibited stable (p>0.05) L* values throughout the storage (Table 1), which could be attributed to muscle pH [19]. The stable pH values, as observed in ORG and NORG samples during storage (Table 1), keep the water holding capacity of meat and its superficial light reflectance, contributing to the maintenance of L* values [19].

In agreement with our results, Fruet et al [18] document ed stable lightness (L* values) in beef from steers finished with concentrate and legume-grass pasture during 13 days of storage. In partial agreement, Kim et al [20] documented similar lightness in meat from pasture-fed and grain-fed elk deer loin during 14 days of storage.

Despite the similarity in lightness ( L* values) in both ORG and NORG samples, NORG steaks exhibited greater positive Delta L* (ΔL* = +1.46) than ORG (ΔL* = 0.00) counterparts (Table 1), which means a lighter appearance of meat.

### Redness (a* value)

**Figure 1:**
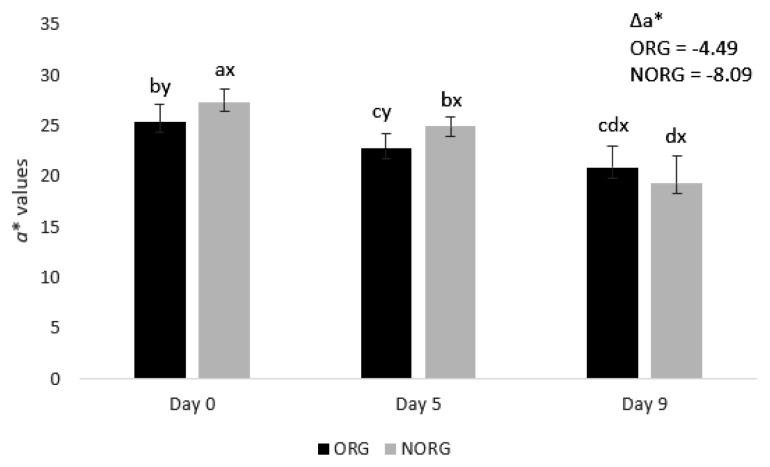
Redness (a* values) and difference of redness (Δa*) of beef *gluteus medius* from cattle raised on organic (ORG) and non-organic (NORG) production systems during aerobic storage at 4°C for 9 days. Standard error bars are indicated. Parameter with production system×storage interaction. ^a–d^ Means within a parameter with different letters are different (p<0.05).^x,y^ Means between production system within a day of storage with different letters are different (p<0.05).

There was a production system×storage interaction (p<0.000; Figure 1) for a* value. NORG steaks exhibited greater (p< 0.05) redness (a* values) than ORG counterparts on days 0 and 5 of storage (Figure 1), whereas ORG and NORG steaks exhibited similar (p>0.05) a* values on day 9 of storage. The observed differences in redness between NORG and ORG steaks may be attributed to changes in muscle energy metabolism [15]. Muscles from grass-fed (such as ORG) cattle usually exhibited less glycolytic metabolism and lactate content than their NORG counterparts, contributing to the decrease of color stability [22] and redness.

Supporting our results, Apaoblaza et al [15] documented higher redness (a* values) in *longissimus dorsi* muscle from grain-fed cattle compared to their forage-fed counterparts. Bjorklund et al [5] observed greater redness in beef from conventional raised (80% concentrate plus 20% forage) Holstein and crossbred steers than in their organic counterparts. In contrast, Revilla et al [17] documented greater redness in *longissimus dorsi* muscle from organic-raised cattle (animals fed 100% on forage) than those raised on feedlot (animals fed with 35% straw fodder and 65% concentrate). Cozzi et al [4] reported greater a* values (redness) in beef from organic pasture-raised cattle than in grain-fed ones.

Both ORG and NORG steaks demonstrated a decrease (p<0.05) in redness (a* values) during storage (Figure 1). The observed decrease in a* values could be attributed to lipid oxidation [23]. The increase of lipid oxidation (as observed in ORG and NORG samples; Table 1) is highly associated with myoglobin oxidation due to the production of free radicals and reactive oxygen species [23], which in turn contributes to the decrease of surface redness (a* values).

In agreement with our results, Lanari et al [24] docu mented a decrease in a* values in GM muscle from pasture and grain-fed cattle during 14 days of storage. In partial agreement, Canto et al [25] reported a decrease in a* values in LL and PM muscle from pasture-fed cattle during 9 days of storage. Salim et al [26] documented a decrease in a* values in PM muscle from grain-fed cattle from day 5 to 9 of storage, whereas the surface redness did not change in LL muscle.

Although NORG samples exhibited overall greater redness (a* values), they also demonstrated greater negative Delta a* (Δa* = −8.09) than ORG counterparts (Δa* = −4.49), which means a more pronounced discoloration (decrease of redness) of NORG samples during storage.

### Yellowness (b* value)

There was a production system×storage interaction (p<0.000; Table 1) for b* value. ORG steaks exhibited greater (p<0.05) yellowness (b* values) than NORG counterparts on day 5 (Table 1), whereas ORG and NORG steaks exhibited similar (p>0.05) b* values on days 0 and 9 of storage. The observed difference in yellowness (b* values) in ORG samples may be attributed to the presence of pigments such as carotenoids, obtained by the grass-feeding regime, contributing to greater deposition of these pigments in meat and consequently increase of b* values [16,21].

In agreement, Cozzi et al [4] observed greater yellowness in beef from organic raised cattle than in their grain-fed counterparts. On contrary, Torrecilhas et al [16] documented similar yellowness (b* values) in *longissimus thoracis* muscle from bulls finished in pasture compared to their feedlot (25:75% corn silage: concentrate) counterparts. Revilla et al [17] evaluated the influence of the production system (conventional vs organic) on beef quality and documented similar yellowness (b* values) in meat from pasture and grain-fed counterparts. Bjorklund et al [5] documented similar b* values (yellowness) in beef from crossbred steers raised under conventional (80% concentrate plus 20% forage) and organic systems.

Both ORG and NORG steaks demonstrated a decrease (p<0.05) in yellowness during storage (Table 1). In contrast, Canto et al [25] reported similar b* values in LL steaks from pasture-fed cattle during 9 days of storage. Salim et al [26] documented similar yellowness (b* values) in LL and PM muscles from grain-fed cattle during 9 days of refrigerated storage.

Regarding Delta, NORG steaks exhibited a greater nega tive Delta b* (Δb* = −3.79) than ORG counterparts (Δa* = −1.47), demonstrating a decrease of yellowness in NORG samples during storage.

### Chroma

**Figure 2:**
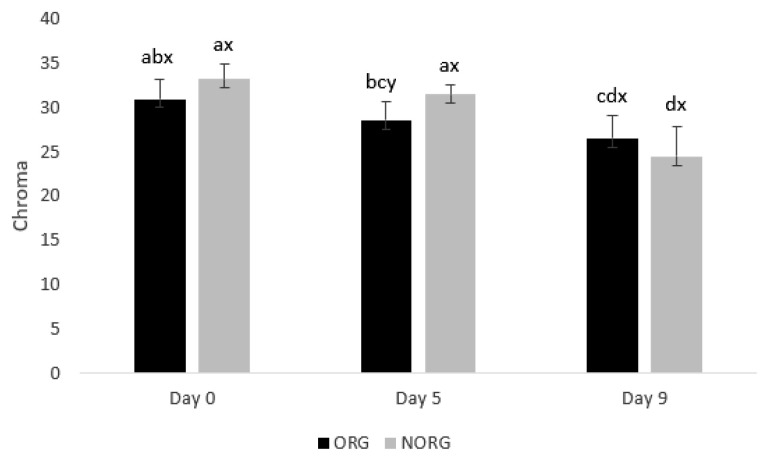
Chroma values of beef *gluteus medius* from cattle raised on organic (ORG) and non-organic (NORG) production systems during aerobic storage at 4°C for 9 days. Standard error bars are indicated. Parameter with production system×storage interaction. ^a–d^ Means within a parameter with different letters are different (p<0.05). ^x,y^ Means between production system within a day of storage with different letters are different (p<0.05).

There was a production system×storage interaction (p = 0.000; Figure 2) for chroma values. NORG steaks exhibited greater (p<0.05) chroma than ORG counterparts on day 5 of storage (Figure 2), whereas ORG and NORG steaks exhibited similar (p>0.05) chroma on days 0 and 9. Chroma expresses how vivid is the color of the meat. Thus, high saturation values mean a more intense red in beef. The observed results could be attributed to the phenolic compounds and flavonoids present in corn [27]. Corn-based diets are rich in phenolic acids, such as ferulic acid and p-coumaric [27], which could exert a protective effect against myoglobin oxidation, contributing to the increase of chroma. A positive correlation (Table 2) was observed between chroma and a* value (r = 0.994; p<0.05), which further reiterates the relationship between these parameters.

In agreement with our results, Morales Gómez et al [28] evaluated the impact of the finishing regime (grain vs pasture) on the color of beef and documented greater chroma in meat from grain-fed cattle than those from pasture-fed on days 0, 7, and 14. Guerrero et al [29] documented greater chroma in beef from grain-fed cattle than in those from pasture-fed on days 1 and 4. In contrast with our results, Salim et al [30] evaluated the influence of feeding systems on the color of beef *longissimus* and reported that muscle from pasture-fed animals tended to have greater chroma (C* values) than those from grain-fed counterparts.

During storage, both ORG and NORG steaks exhibited a decrease (p<0.05) in chroma from day 0 to 9 (Figure 2), which may be attributed to the surface metmyoglobin (MMb) accumulation [31] leading to a brownish appearance of beef.

In agreement, Torrecilhas et al [16] documented a de crease in chroma of both pasture and grain-fed beef from days 4 to 14 of storage. In partial agreement, Guerrero et al [29] documented a decrease in chroma in beef from grain-fed cattle, whereas pasture-fed steaks exhibited stable chroma values during 8 days of storage. Contrasting our results, Morales Gómez et al [28] reported similar chroma of beef from grain-fed and pasture-fed cattle during 14 days of storage.

### Hue angle

**Figure 3:**
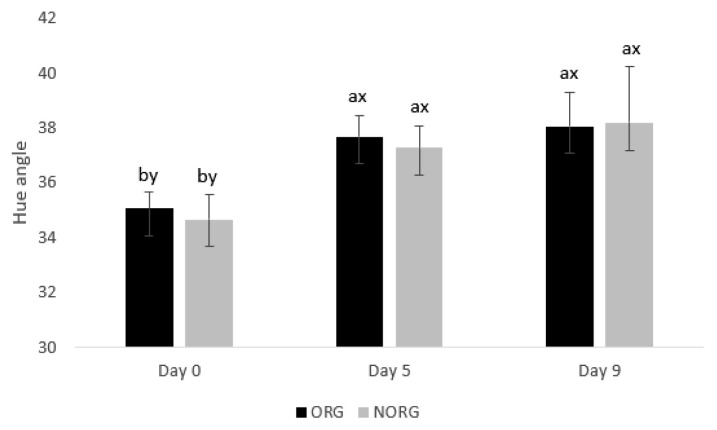
Hue angle of beef *gluteus medius* from cattle raised on organic (ORG) and non-organic (NORG) production systems during aerobic storage at 4°C for 9 days. Standard error bars are indicated. Parameter without production system×storage interaction. ^a,b^ Means within a parameter with different letters are different (p<0.05). ^x,y^ Means between production system within a day of storage with different letters are different (p<0.05).

There was no production system×storage interaction (p = 0.581; Figure 3) for hue angle. However, there was an effect of storage (p = 0.0001) on hue angle. The ORG and NORG steaks exhibited similar (p>0.05) hue angle on days 0, 5, and 9 of storage (Figure 3). The observed similarity in hue values may be attributed to myoglobin content [31]. Hue angle can be expressed as the trueness of red. High values are more intense brown. According to Lindahl et al [31], the pigment (myoglobin) content and the fraction of metmyoglobin on surface color are the most critical factors influencing the variation of hue angle values. In this sense, similar myoglobin contents were documented in ORG (5.33±0.34 mg/g) and NORG (5.91±0.54 mg/g in NORG) samples, which may have contributed to the similarities in hue values.

In partial agreement, Luciano et al [32] documented a similar hue angle in beef *longissimus* from pasture and grain-fed cattle on days 0 and 4 of storage. In contrast with our results, Salim et al [30] reported that muscle from grain-fed animals tended to have greater h* values than the beef from pasture-fed animals on day 0. Lanari et al [24] documented lower hue angle values in GM muscle from pasture-fed cattle than those from grain-fed counterparts from day 4 to 14. Guerrero et al [29] documented a greater hue angle in beef from pasture-fed cattle than in their grain-fed counterparts on days 4 and 8.

Both ORG and NORG exhibited an increase in hue angle values (p<0.05) from day 5 of storage (Figure 3), which could be attributed to myoglobin oxidation and increase of metmyoglobin content on meat surface, which consequently increased hue angle values [31,33]. In agreement with our results, Lanari et al [24] evaluated the influence of pasture feeding or sorghum feeding on beef color and documented an increase in hue angle of pasture and grain-fed GM muscle during 14 days of storage. Luciano et al [32] evaluated the influence of feeding regimes (pasture and grain) on the color of *longissimus* muscle and documented an increase in hue angle from day 0 to 11 of storage. A negative correlation (Table 2) was observed between hue angle and a* value (r = −0.879; p<0.05) and R630/580 (r = −0.978, p<0.05) reiterating the relationship between hue angle, surface redness, and color stability.

### R630/580

**Figure 4:**
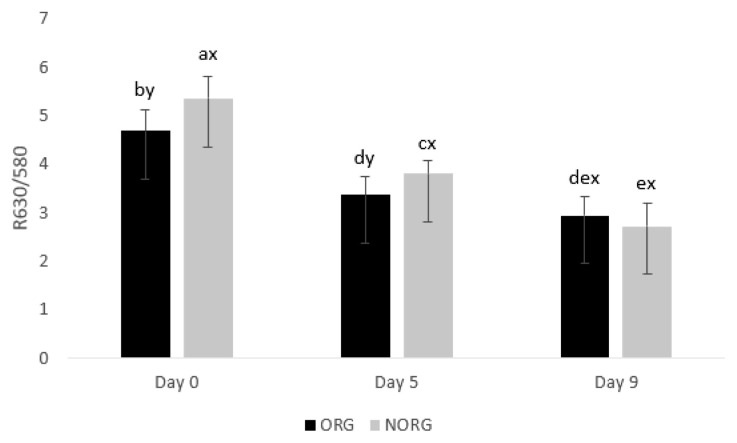
Color stability (R630/580) of beef *gluteus medius* from cattle raised on organic (ORG) and non-organic (NORG) production systems during aerobic storage at 4°C for 9 days. Standard error bars are indicated. Parameter with production system×storage interaction. ^a–e^ Means within a parameter with different letters are different (p<0.05). ^x,y^ Means between production system within a day of storage with different letters are different (p<0.05).

There was a production system×storage interaction (p = 0.000; Figure 4) for R630/580. NORG steaks exhibited greater (p< 0.05) R630/580 than ORG counterparts on days 0 and 5 of storage (Figure 4), whereas ORG and NORG steaks exhibited similar (p>0.05) color stability on day 9 of storage. The ratio of reflectance at 630 nm and 580 nm (R630/580) indirectly estimates the color stability of meat, in which a lower ratio indicates a greater accumulation of metmyoglobin on the beef surface, thus poor color stability or discoloration [9]. In this sense, the observed differences in color stability (R630/580) could be attributed to the phenolic acid intake by grain-fed cattle [27], which delays myoglobin oxidation, contributing to the increase of R630/580. Lipid oxidation exhibited a negative correlation (Table 2) with R630/580 (r = −0.706; p<0.05), a* values (r = −0.726; p<0.05), b* values (r = −0.794; p<0.05) and chroma (r = −0.698; p<0.05); and a positive correlation with hue angle (r = 0.706; p<0.05) reiterating the relationship between these parameters.

In partial agreement, Baldi et al [34] evaluated the influence of feeding regime (grain-based vs forage-based) on color stability of lamb LL and documented similar R630/580 in both grain and forage-based samples on days 1, 2, 3 and 4.

During storage, ORG and NORG steaks demonstrated a decrease (p<0.05) in color stability from day 0 to 9 (Figure 4), which could be attributed to Mb oxidation, mediated by lipid oxidation, and consequently, accumulation of MMb in meat surface [35], decreasing R630/580 values.

In agreement, Baldi et al [34] documented a decrease in R630/580 in lamb LL from grain and forage-based samples during 4 days of storage. Canto et al [25] reported a decrease in R630/580 values in LL and PM muscles from pasture-fed cattle during 9 days of storage. Salim et al [26] documented a decrease in R630/580 in the PM muscle from grain-fed cattle during 9 days of storage, whereas the color stability of LL steaks remained stable.

### Delta E

Delta E expresses the total color change over a period and was calculated from the beginning (day 0) to the end (day 9) of storage. Greater Delta E values reflect greater changes in overall color. NORG steaks exhibited greater (ΔE = 9.05; p<0.05) Delta E than their ORG (ΔE = 4.72; p<0.05) counterparts. The observed difference in beef discoloration may be attributed to the susceptibility of grain-fed samples to oxidative reactions [36] due to their high intramuscular fat content (IMF) [32]. This in turn, can enhance lipid peroxidation, favoring the formation of MMb and concomitant discoloration in NORG samples [37].

### Metmyoglobin reducing activity

**Figure 5:**
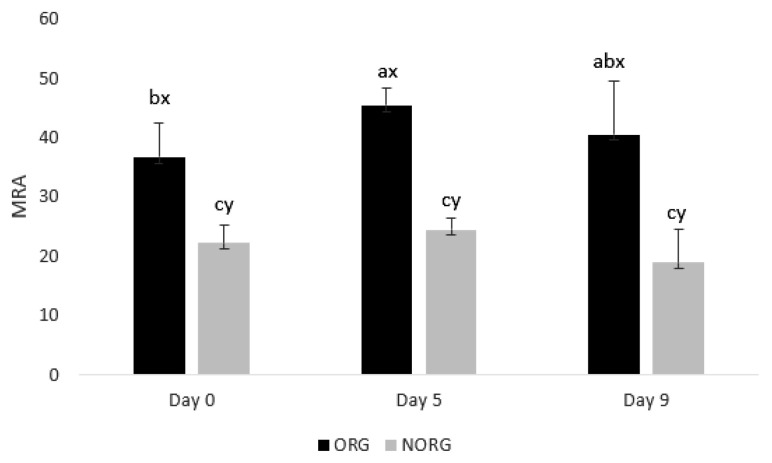
Metmyoglobin reducing activity of beef *gluteus medius* from cattle raised on organic (ORG) and non-organic (NORG) production systems during aerobic storage at 4°C for 9 days. Standard error bars are indicated. Parameter with production system×storage interaction. ^a–c^ Means within a parameter with different letters are different (p<0.05). ^x,y^ Means between production system within a day of storage with different letters are different (p<0.05).

There was a production system×storage interaction (p = 0.012; Figure 5) for MRA. ORG steaks exhibited greater (p<0.05) MRA than NORG counterparts on days 0, 5, and 9 (Figure 5). The observed difference in MRA between ORG and NORG steaks could be attributed to the antioxidant potential of ORG samples [32,36,38].

Metmyoglobin reducing activity is an inherent ability of meat to delay discoloration, associated with the addition of an electron to MMb, via enzymatic or nonenzymatic processes. Nicotinamide adenine dinucleotide (NADH) is an important cofactor involved in MMb reduction, and its regeneration is critical for extending the color stability of meat [39]. In this sense, the meat from pasture-fed animals is associated with high levels of natural antioxidants, such as gallate, catechin, ascorbic acid, α-tocopherol, and β-carotene [36], which act preventing myoglobin oxidation and contributing to the meat color stability [32].

In agreement with our results, Chen et al [38] evaluated the influence of the feeding system (pasture vs. grain) on color stability and antioxidant capacity of LL muscle from yak (*Bos grunniens*) and reported lower metmyoglobin content in LL from pasture-fed yak, than their grain-fed counterparts.

During storage, both ORG and NORG steaks demon strated a stable pattern (p>0.05) in MRA from day 0 to 9 (Figure 5). The observed stability in MRA between ORG and NORG could be attributed to the NADH content in the muscles [22]. NADH is the main component involved in MMb reduction and is regenerated by lactate dehydrogenase in postmortem skeletal muscles [22]. Muscles such as GM had greater enzymatic activity than muscles such as LL contributing to the regeneration of NADH and the maintenance of MRA during storage [40].

In agreement with our results, Salim et al [26] document ed similar MRA in LL muscle from grain-fed cattle during 9 days of refrigerated storage. On the contrary, Canto et al [25] reported a decrease in MRA in LL and PM muscles from pasture-fed cattle for 9 days.

### Lipid oxidation

For lipid oxidation, there was a production system × storage interaction (p = 0.024; Table 1). NORG steaks exhibited greater (p<0.05) lipid oxidation than ORG counterparts throughout the storage (Table 1). The observed differences in lipid oxidation could be attributed to the antioxidant intake from pasture-fed cattle [18], which exerts a protective effect against lipid oxidation [32]. Additionally, the greater lipid oxidation observed in NORG samples could be attributed to a higher IMF [32]. Grain-fed animals are usually heavier and exhibit greater fat content than their pasture-fed counterparts, due to their high energy intakes and lower energy expenditure associated with the feeding regime [21], which could favor the lipid oxidation, increasing TBARS.

In agreement, Torrecilhas et al [16] documented greater lipid oxidation in meat from feedlot cattle than those from pasture ones on day 0. Fruet et al [18] reported higher lipid oxidation and lower α-tocopherol concentration in the meat of bulls finished with grain compared to their pasture-fed counterparts during 13 days of storage. On contrary, Baldi et al [34] documented similar lipid oxidation (TBARS) in LL from grain and forage-fed lambs on days 1 and 4.

ORG and NORG steaks exhibited an increase (p <0.05) in lipid oxidation during storage (Table 1). In this sense, the observed increase in lipid oxidation could be related to a decrease in the redox capacity of meat and the generation of free radicals during storage, which triggers lipid oxidation reactions increasing TBARS [23]. An increase in TBARS has been reported in lamb [34] and beef [18] samples from pasture and grain-fed animals.

### Principal component analysis

**Figure 6:**
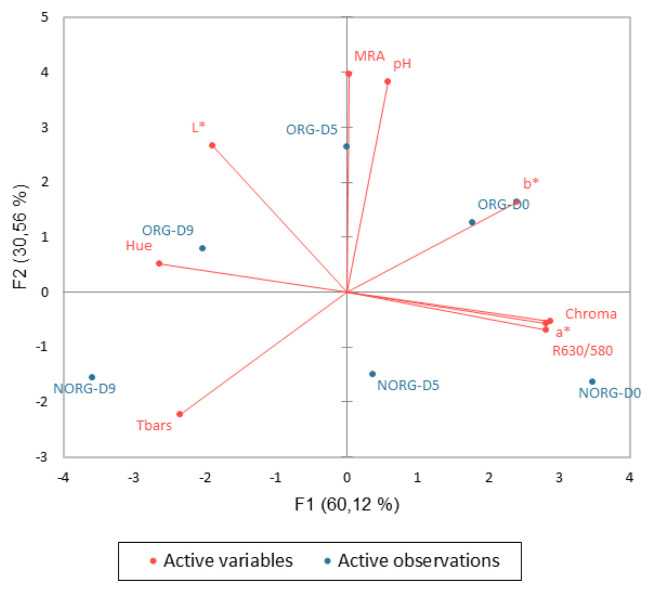
Principal component analysis of physicochemical analysis of beef *gluteus medius* from cattle raised on organic (ORG) and non-organic (NORG) production systems during aerobic storage at 4°C for 9 days.

The PCA explained 90.68% of the total data variance (Figure 6). The first principal component (PC1) contributed to 60.12% of this variance and separated day zero (D0) from day 9 (D9) independent of the production system. Redness (a* values), yellowness (b* values), R630/580, hue angle, chroma, MRA, and TBARS presented square cosines greater than 0.6 and were relevant to this separation. At the beginning of storage (D0), the samples exhibited greater a* values, b* values, R630/580, chroma, and MRA, whereas lower values for lipid oxidation and hue angle than at day 9.

The second principal component (PC2) contributed 30.56% of the variance. This component separated the two production systems based on MRA and pH square cosines. Additionally, the combination of PC1 and PC2 resulted in 4 groups: ORG—D0; NORG—D0 and NORG—D5; ORG—D5 and ORG—D9; and NORG—D9.

## CONCLUSION

The findings of the present study indicated that the production system influenced the quality attributes of GM steaks. ORG steaks had greater pH, yellowness, and metmyoglobin reducing activity, whereas NORG steaks exhibited greater redness, chroma, color stability (R630/580), and lipid oxidation than their ORG counterparts. Although NORG samples exhibited better-appearing color, they exhibited greater Delta E, which means greater discoloration than their ORG counterparts during storage. Therefore, the production system affects beef color stability and lipid oxidation during refrigerated storage.

## Figures and Tables

**Table 1:** Lightness (L*values), the difference of lightness (ΔL*), yellowness (b* values), the difference of yellowness (Δb*) meat pH, and lipid oxidation of beef *gluteus medius* from cattle raised on organic (ORG) and non-organic (NORG) production systems during aerobic storage at 4°C for 9 days

Parameter	Production system	Days of storage			Delta

		0	5	9	
L* value	ORG	42.38±1.79^ax^	42.22±1.68^ax^	42.38±1.87^ax^	ΔL* = 0.00
	NORG	40.77±2.54^ax^	41.42±2.25^ax^	42.23±3.24^ax^	ΔL* = +1.46
b* value^1)^	ORG	17.76±1.32^abx^	19.19±0.66^ax^	16.29±1.69^bcx^	Δb* = −1.47
	NORG	18.88±1.12^ax^	17.28±1.46^by^	15.09±2.03^cx^	Δb* = −3.79
Meat pH^1)^	ORG	5.60±0.06^abx^	5.67±0.13^ax^	5.52±0.07^bcx^	
	NORG	5.50±0.07^cy^	5.47±0.06^cy^	5.48±0.08^cx^	
Lipid oxidation^1),2)^	ORG	0.007±0.002^dx^	0.018±0.004^cdy^	0.026±0.005^bcy^	
	NORG	0.016±0.003^cdx^	0.031±0.007^bx^	0.052±0.010^ax^	

Results are expressed as average±standard deviation (SD).

1) Parameters with production system×storage interaction.

2) Result expressed as absorbance at 532 nm.

a–d Means within a parameter with different letters are different (p<0.05).

x,y Means between production system within a day of storage with different letters are different (p<0.05).

**Table 2:** Correlation matrix lightness (L* values), redness (a* values), yellowness (b* values), color stability (R630/580), hue angle, chroma, metmyoglobin reducing activity, lipid oxidation (TBARS), and meat pH

Variables	L* values	a* values	b* values	R630/580	Hue angle	Chroma	MRA	TBARS	pH
L* values	**1**	−0.706	−0.421	−0.661	0.528	−0.722	0.583	0.112	0.461
a* values		**1**	0.747	**0.953**	**−0.879**	**0.994**	−0.104	−0.726	0.057
b* values			**1**	0.659	−0.567	0.744	0.387	−0.794	0.595
R630/580				**1**	**−0.978**	**0.916**	−0.182	−0.706	0.037
Hue angle					**1**	**−0.825**	0.164	0.706	−0.072
Chroma						**1**	−0.101	−0.698	0.032
MRA							**1**	−0.563	**0.828**
TBARS								**1**	−0.600
pH									**1**

MRA, metmyoglobin reducing activity; TBARS, thiobarbituric acid reactive substances.
